# Supplementary material for: Cellular encoding of Cy dyes for single-molecule imaging
Source: eLife. 2016 Dec 12;5:e19088. doi: 10.7554/eLife.19088 (PMC5207767; doi:10.7554/eLife.19088)
Supplement: Supplementary file 1. — In total, Xenopus laevis oocytes of stage V and VI express 1300 genes (see Materials and methods) and 18% of those (234 genes) contain TAG as a stop codon. 43 (shown here) are predicted to be membrane proteins, roughly half of which are categorized as mitochondrial proteins or proteins with unknown function. DOI: http://dx.doi.org/10.7554/eLife.19088.017 [file elife-19088-supp1.docx]

| Unigene  ID | Protein ID | Gene ID | Protein  length | Gene  length | Protein Name |
| --- | --- | --- | --- | --- | --- |
| Xl.412 | NP_001084206.1 | NM_001090737.1 | 583 | 1752 | frizzled class receptor 8 precursor |
| Xl.2879 | NP_001080323.1 | NM_001086854.1 | 147 | 444 | mitochondrial import receptor subunit TOM20 homolog |
| Xl.3214 | NP_001086011.1 | NM_001092542.1 | 362 | 1089 | MGC83607 protein |
| Xl.3881 | NP_001080371.1 | NM_001086902.1 | 290 | 873 | elongation of very long chain fatty acids-like 1 |
| Xl.4072 | XP_002940168.1 | XM_002940122.1 | 964 | 2895 | PREDICTED: n-alpha-acetyltransferase 25, NatB auxiliary subunit |
| Xl.4285 | NP_001091224.1 | NM_001097755.1 | 434 | 1305 | transmembrane protein 194A precursor |
| Xl.6484 | NP_001086502.1 | NM_001093033.1 | 643 | 1932 | mannosidase, alpha, class 1A, member 2 |
| Xl.7097 | NP_001090209.1 | NM_001096740.1 | 460 | 1383 | serine incorporator 1 precursor |
| Xl.7137 | XP_002936931.1 | XM_002936885.1 | 107 | 324 | PREDICTED: hypothetical protein LOC100379706 |
| Xl.12577 | NP_001091435.1 | NM_001097966.1 | 336 | 1011 | uncharacterized protein LOC100049138 |
| Xl.12727 | NP_001082707.1 | NM_001089238.1 | 369 | 1110 | fatty acid 2-hydroxylase |
| Xl.14572 | XP_002941767.1 | XM_002941721.1 | 185 | 558 | PREDICTED: hypothetical protein LOC100487322 |
| Xl.15165 | NP_001089717.1 | NM_001096248.1 | 354 | 1065 | uncharacterized protein LOC734780 |
| Xl.19877 | NP_001080748.1 | NM_001087279.1 | 309 | 930 | F-box and leucine-rich repeat protein 5 |
| Xl.22300 | NP_001085946.1 | NM_001092477.1 | 373 | 1122 | DnaJ (Hsp40) homolog, subfamily B, member 12 |
| Xl.23657 | NP_008139.1 | NC_001573.1 | 226 | 1140 | ATP synthase F0 subunit 6 (mitochondrion) |
| Xl.23896 | NP_001086386.1 | NM_001092917.1 | 174 | 525 | cytochrome c oxidase subunit IV isoform 2 (lung) |
| Xl.25760 | NP_001086574.1 | NM_001093105.1 | 331 | 996 | transmembrane protein 171 |
| Xl.29182 | XP_002938502.1 | XM_002938456.2 | 404 | 1215 | PREDICTED: trophoblast glycoprotein |
| Xl.31091 | NP_008135.1 | NC_001573.1 | 345 | 1140 | NADH dehydrogenase subunit 2 (mitochondrion) |
| Xl.32693 | NP_001088269.1 | NM_001094800.1 | 335 | 1008 | erlin-2-A precursor |
| Xl.33070 | NP_001089785.1 | NM_001096316.1 | 138 | 417 | golgi transport 1B |
| Xl.42475 | NP_008140.1 | NC_001573.1 | 260 | 1140 | cytochrome c oxidase subunit III (mitochondrion) |
| Xl.45105 | NP_001084619.1 | NM_001091150.1 | 318 | 957 | COP9 signalosome complex subunit 6 |
| Xl.45218 | NP_001090043.1 | NM_001096574.1 | 213 | 642 | transmembrane protein 52 precursor |
| Xl.45565 | NP_001084683.1 | NM_001091214.1 | 345 | 1038 | Novel 7 transmembrane receptor (rhodopsin family) protein |
| Xl.45628 | NP_001083670.1 | NM_001090201.1 | 1306 | 3921 | adhesion G protein-coupled receptor A2 precursor |
| Xl.48396 | NP_001086891.1 | NM_001093422.1 | 684 | 2055 | solute carrier family 5 (sodium/iodide cotransporter), member 5 |
| Xl.52407 | XP_002938878.1 | XM_002938832.1 | 1021 | 3066 | PREDICTED: LOW QUALITY PROTEIN: uncharacterized protein KIAA0319-like |
| Xl.53287 | NP_008136.1 | NC_001573.1 | 518 | 1140 | cytochrome c oxidase subunit I (mitochondrion) |
| Xl.53620 | NP_008137.1 | NC_001573.1 | 229 | 1140 | cytochrome c oxidase subunit II (mitochondrion) |
| Xl.55827 | NP_001089260.1 | NM_001095791.1 | 872 | 2619 | extended synaptotagmin-2-A |
| Xl.61927 | NP_001085872.1 | NM_001092403.1 | 103 | 312 | MGC80968 protein |
| Xl.66309 | NP_001086011.1 | NM_001092542.1 | 362 | 1089 | MGC83607 protein |
| Xl.71057 | NP_001106304.1 | NM_001112833.1 | 347 | 1044 | coxsackie virus and adenovirus receptor precursor |
| Xl.75886 | NP_008134.1 | NC_001573.1 | 323 | 1140 | NADH dehydrogenase subunit 1 (mitochondrion) |
| Xl.76239 | NP_008146.1 | NC_001573.1 | 379 | 1140 | cytochrome b (mitochondrion) |
| Xl.76372 | NP_008144.1 | NC_001573.1 | 604 | 1140 | NADH dehydrogenase subunit 5 (mitochondrion) |
| Xl.76806 | NP_001079587.1 | NM_001086118.1 | 139 | 420 | Selenoprotein T-like |
| Xl.76933 | NP_008141.1 | NC_001573.1 | 114 | 1140 | NADH dehydrogenase subunit 3 (mitochondrion) |
| Xl.77528 | XP_002940168.1 | XM_002940122.1 | 964 | 2895 | PREDICTED: n-alpha-acetyltransferase 25, NatB auxiliary subunit |
| Xl.77690 | NP_001079587.1 | NM_001086118.1 | 139 | 420 | Selenoprotein T-like |
| Xl.85149 | NP_001087580.1 | NM_001094111.1 | 552 | 1659 | diacylglycerol kinase, epsilon 64kDa |
